# A Case Series of Familial Macular Drusen: Clinical, Imaging, and Diagnostic Considerations in the Spectrum Between Age-Related Macular Degeneration (AMD) and Other Macular Disorders

**DOI:** 10.7759/cureus.105478

**Published:** 2026-03-19

**Authors:** Giovani Faustine, Mae-Lynn C Bastion

**Affiliations:** 1 Department of Ophthalmology, National University of Malaysia, Kuala Lumpur, MYS

**Keywords:** age-related macular degeneration, amd, drusen, hereditary macular disorders, south-east asian

## Abstract

Familial clustering of macular drusen in younger adults can mimic age-related macular degeneration (AMD) but may reflect an underlying inherited macular disorder. We describe a 72-year-old Chinese woman with high myopia and advanced neovascular AMD in one eye, who presented with progressive visual decline. Despite anti-vascular endothelial growth factor therapy, vision remained poor. Concerned about familial risk, her three adult children were examined. All three adult children (aged 46, 38, and 36 years) were asymptomatic despite drusen being detected on examination. This multigenerational clustering of drusen at relatively young ages raises suspicion for a possible hereditary macular disorder. This case highlights the need for family screening and genetic evaluation in atypical AMD presentations.

## Introduction

Age-related macular degeneration (AMD) is usually a sporadic, late-onset disease that commonly presents as a multifactorial condition affecting individuals older than 60 years old [1]. However, several inherited macular dystrophies, including autosomal dominant drusen and vitelliform dystrophies, may clinically resemble AMD and present with drusen at a younger age, which are often overlooked [2,3]. We report a Chinese family in which the index patient had presumed advanced AMD, while all three adult children demonstrated macular drusen. The presence of drusen in younger first-degree relatives raises the possibility of an underlying hereditary macular dystrophy rather than sporadic AMD. This case series highlights the importance of family evaluation in apparent AMD cases and discusses the differential diagnosis between age-related and inherited macular disorders.

## Case presentation

Index patient

A 72-year-old Chinese woman with a background of hypertension and dyslipidemia presented with a longstanding history of visual deterioration in both eyes. She reported blurred vision in her right eye for the past 11 years and a more recent decline in the left eye over a year. Ocular history was notable for bilateral pseudophakia, high myopia, and prior intravitreal injections elsewhere.

**Figure 1:**
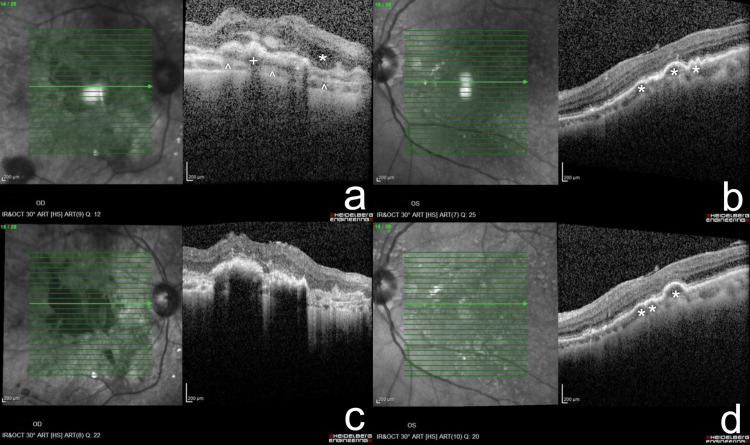
OCT findings of the index patient Index patient initial OCT shows thickening and elevation of RPE (^), intraretinal (*) and subretinal (+) fluid, and macular scarring suggestive of neovascular AMD in the right eye (a) with drusen (*) in the left eye (b). Follow-up OCT in the subsequent years demonstrated persistent disciform scarring and subretinal changes in the right eye (c), with progression of drusen (*) forming pigment epithelium detachments suggestive of exudative AMD development in the left eye (d). Original clinical images. OCT: Optical coherence tomography; AMD: age-related macular degeneration; RPE: retinal pigment epithelium

On examination, her right eye demonstrated a best-corrected visual acuity of hand movement and 6/15 for distance and N12 for near in the left eye. Anterior segment examination on both eyes was unremarkable. Fundus examination shows evidence of disciform scarring involving the macula of the right eye and the presence of confluent soft drusen at the macula of the left eye. No other fundus abnormalities noted. Optical coherence tomography (OCT) revealed features consistent with advanced neovascular AMD in the right eye and high-risk dry AMD in the left eye (Figures 1a, 1b). She had previously undergone treatment with intravitreal bevacizumab (Avastin®), in addition to topical artificial tears and daily oral antioxidant supplementation (Vitalux®). Despite therapy, follow-up OCT demonstrated persistent macular scarring with no resolution of subretinal fluid (Figures 1c, 1d), and visual acuity remained limited to hand movement. This led her to express concern about a potential familial component to her condition and to request her children undergo ocular evaluation.

Family evaluation

**Figure 2:**
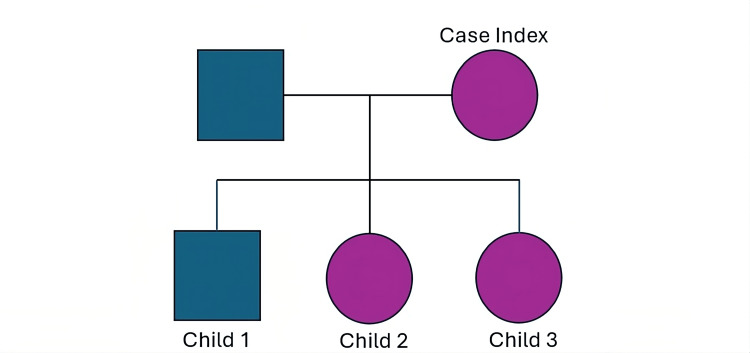
Pedigree of the family showing the index case (mother) and her three children All three offspring demonstrated macular drusen on examination, supporting a possible autosomal dominant inheritance pattern. Original illustration.

**Figure 3:**
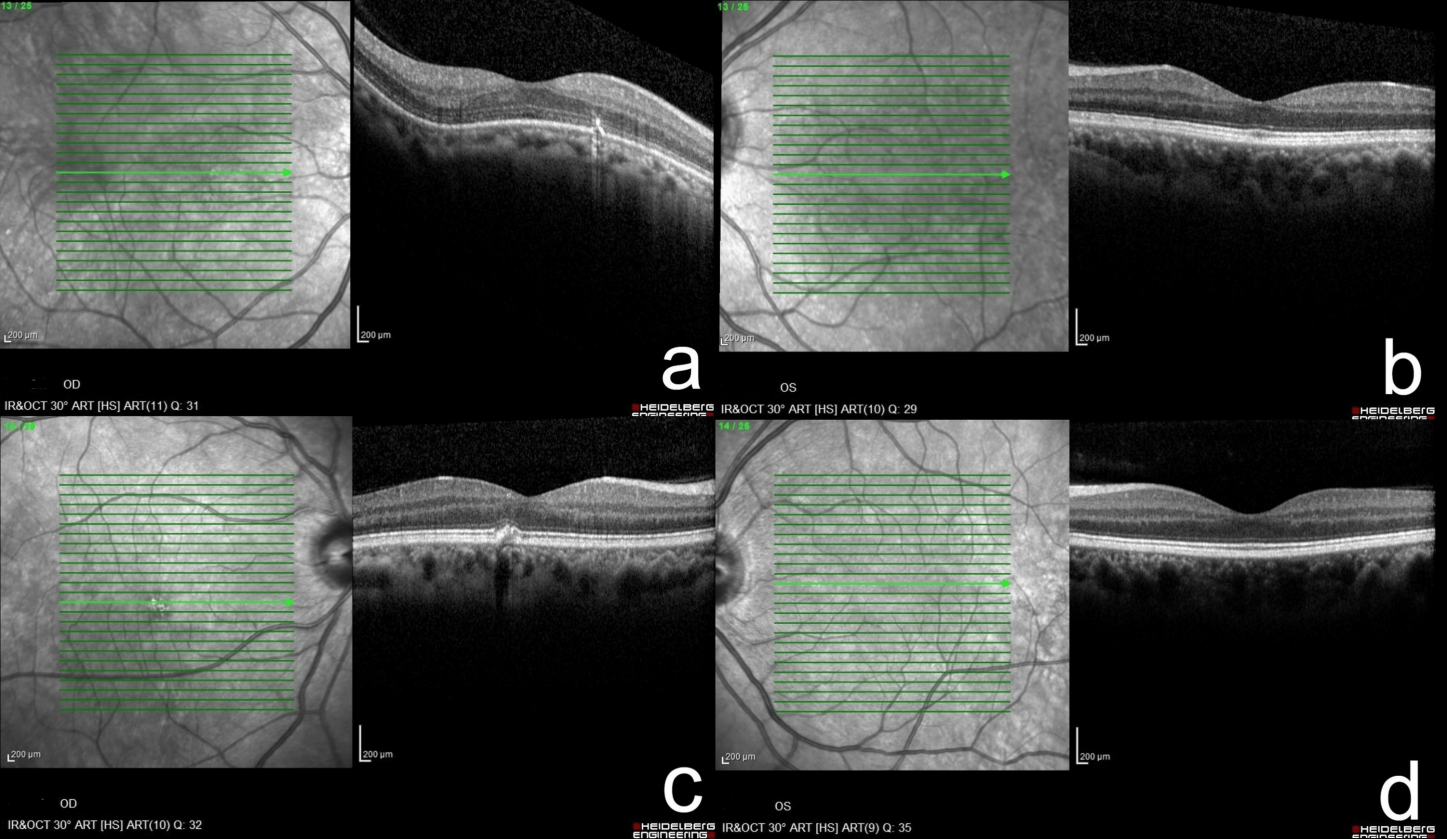
OCT findings of the first child and the second child Child 1 (46-year-old man) OCT shows a single druse near the fovea on the right eye (a) with preserved foveal contour on the left eye (b). Child 2 (38-year-old woman) OCT shows multiple soft drusen over the fovea of the right eye (c) and no druse with preserved foveal contour on the left eye (d). Original clinical images. OCT: Optical coherence tomography

All three of the patient’s children, as illustrated in the family pedigree (Figure 2), underwent ophthalmic examination as part of family screening. There was no known parental consanguinity. Her eldest child, a 46-year-old male, had a known history of amblyopia in the right eye since childhood and wore myopic corrective lenses. He was otherwise medically well and asymptomatic. Fundus examination revealed a single druse in right eye macula. Serial OCT scans over three years showed no significant changes (Figures 3a, 3b).

The second child, a 38-year-old woman, also reported no systemic illnesses and had been using myopic glasses. She was similarly asymptomatic, but fundus examination and OCT revealed multiple soft drusen on the right eye (Figure 3c, 3d).

**Figure 4:**
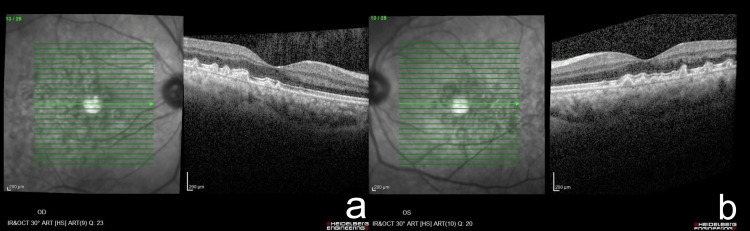
OCT finding of the last child Child 3 (36-year-old woman) OCT shows multiple bilateral soft drusen over both right (a) and left (b) eyes. Original clinical images. OCT: Optical coherence tomography

The youngest child, a 36-year-old woman, shared a similar background of myopia but was otherwise systemically and visually asymptomatic. However, examination revealed multiple soft drusen in both eyes with stable findings on annual follow-ups (Figure 4). In light of these findings, all were advised to undergo regular six-monthly ophthalmic follow-up and instructed to monitor for early signs of visual distortion using an Amsler grid at home. Clinical and imaging findings for all patients are summarized in Table 1.

**Table 1:** Short summary of patients and their findings AMD: Age-related macular degeneration; OCT: optical coherence tomography

Patient	Fundus and OCT Findings
Index patient (Mother), 72-year-old woman	RE disciform macular scar, advanced neovascular AMD with macular scarring LE confluent soft drusen, high-risk dry AMD
Child 1 (Eldest), 46-year-old man	Single soft druse on the right macula
Child 2 (Middle), 38-year-old woman	Multiple soft drusen on the right macula
Child 3 (Youngest), 36-year-old woman	Multiple soft drusen on both eyes

Although none of the children had reported symptoms or decline in visual acuity, the presence of macular changes among first-degree relatives (Figure 2) may suggest a possible hereditary component. These findings support continued clinical monitoring and consideration of genetic counselling where appropriate.

## Discussion

AMD is characterized by the accumulation of drusen between the retinal pigment epithelium (RPE) and Bruch’s membrane [4]. It is among the most prevalent retinal diseases worldwide, affecting approximately 1-3% of the general population, with prevalence rising to nearly 10% in individuals over 65 years and up to 25% in those older than 75 in resource-rich countries [1]. Non-modifiable risk factors associated with AMD include female sex, family history of AMD, hyperopia, and ethnicity. Prevalence has been reported to be highest among White individuals (5.4%), followed by Asians (4.6%), Hispanics (4.2%), and African Americans (2.4%) [5,6]. Genetic variants such as homozygous complement factor H (CFH) Y402H polymorphism or ARMS2 A69S polymorphism have shown consistent increased risk across both Western and Asian populations. A study in a Southeast Asian cohort further supports ARMS2 del443ins54 and ARMS2 A69S polymorphisms as significant contributors to neovascular AMD susceptibility [7]. This data may underrepresent Southeast Asian populations, underscoring the importance of reporting atypical familial cases.

While AMD typically occurs after age 60, a study of multiplex AMD families demonstrated that carriers of rare complement gene variants developed symptoms approximately four years earlier than noncarriers, highlighting the influence of genetic predisposition [8]. The index patient in our case falls within the typical age bracket for disease onset. However, the presence of bilateral disease with poor response to therapy in the index patient, along with all three adult children, demonstrated macular drusen at ages that were considerably younger than expected for conventional AMD, distinguishing this family from most population-level AMD cases.

Another consideration is adult-onset foveomacular vitelliform dystrophy (AOFVD), an autosomal dominant, slowly progressive macular dystrophy that typically presents between the third and fifth decades of life and has been associated with mutations in the *PRPH2* or *BEST1 *genes [2]. Spectral-domain OCT characteristically reveals a hyperreflective dome-shaped vitelliform lesion between the RPE and the neurosensory retina that might appear homogenous or heterogeneous and may leave a hyporeflective cavitation [2,9]. On fundus autofluorescence, these lesions show increased autofluorescence, while fluorescein angiography often demonstrates hypofluorescent lesions surrounded by irregular annular hyperfluorescence, with late staining of vitelliform material [2].

Unlike AMD, increased subfoveal choroidal thickness is often observed in AOFVD [9]. In our series, these specific imaging features were not evident on available OCT, and multimodal imaging was not performed, which limits our ability to confidently include or exclude AOFVD as a diagnosis. Nonetheless, a possible autosomal dominant pattern with early macular involvement remains an important diagnostic consideration.

Familial dominant drusen, including Malattia Leventinese/Doyne honeycomb retinal dystrophy, is characterized by early-onset drusen in a radial or honeycomb pattern and is frequently associated with EFEMP1 mutations [3]. The early appearance of drusen in the patient’s children may suggest this entity, yet the clinical phenotype in this family does not fully conform to the typical description. Drusen-like deposits can also be seen in some systemic disorders [10,11]; however, no clinical history suggestive of these conditions was reported in this family.

Limitations

This case report is limited by the absence of confirmatory genetic characterization, lack of multimodal imaging or angiographic confirmation. However, the phenotypic findings did not clearly indicate a single candidate dystrophy to justify targeted gene analysis, and broad untargeted genetic screening would impose substantial cost with uncertain clinical yield. In highly specialized centers with access to expanded retinal dystrophy panels or research-based sequencing, further genetic evaluation may be considered to better delineate the underlying condition. The family continues to undergo longitudinal clinical and imaging surveillance, which remains a pragmatic approach in evolving or indeterminate hereditary macular phenotypes.

## Conclusions

This case highlights the diagnostic complexity of macular degeneration across multiple generations with early-onset features that suggests a hereditary etiology beyond sporadic AMD. The clinical findings did not fully align with any single well-characterized dystrophy, and the absence of multimodal imaging and genetic testing limits definitive classification. Nevertheless, the familial clustering and consistent macular changes support continued surveillance, patient education, and consideration of referral for genetic counselling, with targeted or panel-based testing guided by future phenotypic clarification. Further study may improve understanding of hereditary macular disorders in underrepresented ethnic populations.

## References

[1] Wong WL, Su X, Li X, Cheung CM, Klein R, Cheng CY, Wong TY (2014). Global prevalence of age-related macular degeneration and disease burden projection for 2020 and 2040: a systematic review and meta-analysis. Lancet Glob Health.

[2] Nipp GE, Lee T, Sarici K, Malek G, Hadziahmetovic M (2023). Adult-onset foveomacular vitelliform dystrophy: epidemiology, pathophysiology, imaging, and prognosis. Front Ophthalmol (Lausanne).

[3] de Guimarães TA, Kalitzeos A, Mahroo OA, van der Spuy J, Webster AR, Michaelides M (2024). A long-term retrospective natural history study of EFEMP1-associated autosomal dominant drusen. Invest Ophthalmol Vis Sci.

[4] VanDenLangenberg AM, Carson MP (2025). Drusen bodies. StatPearls [Internet].

[5] Kodjikian L, Rezkallah A, Decullier E, Aulagner G, Huot L, Mathis T (2005). Early predictive factors of visual loss at 1 year in neovascular age-related macular degeneration under anti-vascular endothelial growth factor. Ophthalmol Ret.

[6] Klein R, Klein BE, Knudtson MD (2006). Prevalence of age-related macular degeneration in 4 racial/ethnic groups in the multi-ethnic study of atherosclerosis. Ophthalmology.

[7] Supanji S, Romdhoniyyah DF, Sasongko MB (2021). Associations of ARMS2 and CFH gene polymorphisms with neovascular age-related macular degeneration. Clin Ophthalmol.

[8] Helgason H, Sulem P, Duvvari MR (2013). A rare nonsynonymous sequence variant in C3 is associated with high risk of age-related macular degeneration. Nat Genet.

[9] Murthy RK, Haji S, Sambhav K, Grover S, Chalam KV (2016). Clinical applications of spectral domain optical coherence tomography in retinal diseases. Biomed J.

[10] Sen S (2021). Drusen-like deposits in systemic disorders: a point of convergence for nephrologists and ophthalmologists. J Postgrad Med.

[11] Khan KN, Mahroo OA, Khan RS (2016). Differentiating drusen: Drusen and drusen-like appearances associated with ageing, age-related macular degeneration, inherited eye disease and other pathological processes. Prog Retin Eye Res.

